# Early-Stage Non-Small Cell Lung Cancer Stereotactic Body Radiation Therapy (SBRT) Outcomes in an Equal Access Military Setting

**DOI:** 10.7759/cureus.13485

**Published:** 2021-02-22

**Authors:** Avinash R Chaurasia, John White, Robert C Beckmann, Michael Chamberlin, Adam Horn, Anna M Torgeson, William Skinner, Delnora Erickson, Aaron Reed

**Affiliations:** 1 Radiation Oncology, National Capital Consortium, Bethesda, USA; 2 Radiation Oncology Residency, National Capital Consortium, Bethesda, USA; 3 Radiation Oncology, Tripler Army Medical Center, Honolulu, USA; 4 Radiation Oncology, Naval Medical Center, San Diego, USA; 5 Radiation Oncology, Chesapeake Urology, Gaithersburg, USA

**Keywords:** lung cancer, non-small cell lung cancer, radiation therapy, stereotactic body radiation therapy, stereotactic ablative body radiotherapy, community, equal access, military

## Abstract

Introduction

Lung stereotactic body radiation therapy (SBRT) is a first-line treatment for early-stage lung cancer in non-surgical candidates or those who refuse surgery. We compared our institutional outcomes from a unique patient population with decreased barriers to care with a recently published prospective series.

Materials and methods

We retrospectively reviewed all patients who received definitive lung SBRT at the Walter Reed National Military Medical Center from 2015 to 2020. All patients underwent a positron emission tomography-computed tomography (PET-CT) and all were presented at a multidisciplinary tumor board. Patients were treated on a Trubeam linear accelerator (LINAC)-based system with daily cone-beam CT. The results were qualitatively compared to outcomes from prospective studies including RTOG 0236 and RTOG 0618.

Results

A total of 105 patients with 114 lesions were included. Median age was 77 years and 54.7% had ≥ 40-pack year smoking history. 36.8% did not have pathologic confirmation. With a median follow-up of 24 months, three-year local control (LC), disease-free survival (DFS) and overall survival (OS) rates were 92.4%, 81.0%, and 80.0%, respectively. Rates of Grade 1 and 2 toxicity were 21.9% and 6.7% and no patients experienced Grade ≥ 3 toxicity.

Conclusions

In our military setting with universal coverage and routine multidisciplinary care, lung SBRT provides outcomes comparable to prospective studies conducted at high-volume academic centers. More than one-third of patients were treated empirically without pathologic confirmation of disease, demonstrating a difference between clinical trials and community practice. Further investigation is warranted to integrate multidisciplinary management and achieve equal access to care to bridge existing health disparities in the community setting.

## Introduction

Compared to conventionally fractionated radiation therapy, lung stereotactic body radiation therapy (SBRT) has demonstrated improved rates of local control (LC) without increasing the risk of major toxicity [1,2]. High-quality data from multiple Radiation Therapy Oncology Group (RTOG)/NRG Oncology trials have confirmed the effectiveness and safety of SBRT in a controlled population of patients [3,4]. As a result, SBRT is now recommended as a standard therapy for early-stage non-small cell lung cancer (NSCLC) who are not surgical candidates or for patients who refuse surgery. In response to these reports, smaller community-based cancer centers have developed the technical expertise required to plan and deliver high-quality SBRT and this technique has rapidly expanded to become a routine part of clinical practice.

While prospective and other retrospective studies illustrate the success of SBRT at high volume academic centers, its generalizability to smaller community practice is not well established. Multiple studies of patients across all stages of lung cancer treated at community centers have decreased access to the latest breakthroughs in treatment which may lead to decreased survival outcomes [5,6]. Aside from the technical requirements, patients may face other significant barriers to care in the community setting that can portend for decreased lung cancer survival: lower rates of primary care, geographical difficulties to seeking care, and sociodemographic divides [7,8]. Patients in community centers are also less likely to receive SBRT as compared to the academic setting [7,9].

The United States military health system (MHS) represents a unique patient population with universal access to care for our beneficiaries. Our center began to integrate regular lung cancer screening for high-risk patients in 2014, leading to higher numbers of early-stage lung cancer identification. In the two-year span of 2013-2014, our clinic treated only six patients with SBRT. However, in the period from 2016 to 2017, we treated a total of 49 patients with 58 lesions. Compared to the community, our patients may have decreased barriers to care such as universal coverage, reliable follow-up patterns, and well-integrated multidisciplinary care. Additionally, there have been reports that show that lung cancer patients in the MHS demonstrate improved survival compared to large national registries, suggesting that there may be improved survival among patients in this unique equal access system [10].

In this study, we compared our institutional outcomes for early-stage NSCLC treated with definitive SBRT to recently published prospective data in order to determine the generalizability to our unique military setting with equal access to care.

## Materials and methods

We retrospectively reviewed all patients who were treated with definitive lung SBRT for early-stage NSCLC at our institution (Walter Reed National Military Medical Center; WRNMMC) from January 2015 to August 2020 after obtaining Institutional Review Board (IRB) approval. Patient demographic, treatment characteristics and outcome data were collected for each patient. All patients were presented at multidisciplinary tumor board including Cardiothoracic Surgery, General & Interventional Pulmonology, Medical Oncology, Radiology, Pathology, and Radiation Oncology. This tumor board met weekly and reviewed patients at initial diagnosis and following any changes to their clinical status. Any decision to proceed with SBRT without biopsy confirmation of disease was communicated and agreed upon in this forum, typically based on the predicted probability of malignancy (i.e., enlarging PET-avid lesion on serial scans) weighed against risks of biopsy.

PET-CT was obtained in all patients for staging and target delineation. A 4D-CT simulation was utilized and planning was done on the CT average. An initial gross target volume (GTV) was contoured and then expanded to an internal target volume (ITV) for motion management. This was then expanded 5 millimeters to a planning target volume (PTV).

Patients were treated with linear accelerator (LINAC)-based SBRT. Image guidance was accomplished through daily cone-beam CT scans without fiducial assistance (per institutional practice). The most commonly used dose/fractionation was 50 Gy in 5 fractions prescribed to the PTV.

All patients were followed with surveillance CT imaging at initial follow-up three months post-SBRT, with CT scans performed thereafter every three to six months. Any suspicious findings on subsequent CT scans would prompt another PET-CT to further investigate potential disease recurrence vs post-treatment changes. Biopsies were considered when either the CT or the PET-CT suggested progression or recurrence. These decisions were also made in a multidisciplinary setting, as noted above.

Recurrence was recorded according to local (within high dose volume), lobar, regional, locoregional (including mediastinal), or disseminated. LC was defined as no recurrence within the high dose region or within the same lung lobe. LC, disease-free survival (DFS) and overall survival (OS) were recorded and calculated using the Kaplan-Meier method. LC was analyzed for each individual tumor (some patients had > 1 treated tumor), whereas DFS and OS were calculated for each patient. Treatment toxicity was recorded according to the Common Terminology Criteria for Adverse Events (CTCAE) 5.0.

The results were qualitatively compared to outcomes presented in the RTOG 0236 and RTOG 0618.

## Results

From January 2015 to August 2020, 105 patients with a median follow-up of 23.5 months (range 0-62 months) were analyzed. The median age at treatment was 77 years old, and the median pack-year of smoking was 40. The breakdown of treated lesion size by AJCC 8th edition T-stage for T1, T2, and T3 was 72.8%, 20.2%, and 3.5%, respectively. Median lesion size was 1.7 cm and median PET SUV was 3.93. 86.0% of patients received 50 Gy in 5 fractions (BED10 = 100.00Gy), 3.5% 54 Gy in 3 fractions (BED10 = 151.20), and 10.5% of patients received 60 Gy in 8 fractions (BED10 = 105.00Gy). These data are summarized in Table 1.

 

**Table 1:** Patient characteristics. pk/yr: pack-years; PFTs: pulmonary function tests; FEV1: forced expiratory volume in one second; DLCO: diffusing capacity for carbon monoxide; SCC: squamous cell carcinoma; PTV: planning target volume; fx: fractions; OAR: organ-at-risk; ITV: internal target volume.

Sex	n	%
Male	60	57.1
Female	45	42.8
Age	Median	Range
(years)	77	(56-90)
Smoking	n	%
<10 pk/yr	19	17.9
10-39 pk/yr	22	20.8
40 or more pk/yr	58	54.7
PFTs (%)	Median	Range
FEV 1	79	(30-116)
DLCO	57	(30-122)
Stage	n	%
T1	83	72.8
T2	23	20.2
T3	11	3.5
Size	Median	Range
(cm)	1.7	(0.7-5.5)
Histology	n	%
Adenocarcinoma	49	43
SCC	24	21.1
Carcinoid/neuroendocrine	2	1.8
No biopsy	26	22.8
Indeterminate	13	11.4
PTV	Median	Range
(cc)	22.1	(5.6-168.7)
Dose/# fractions	n	%
50 Gy/ 5 fx	98	86
54 Gy/ 3 fx	4	3.5
60 Gy/ 8 fx	12	10.5
Dmax	Median	Range
(%)	123.6	(93.3-147.0)
OARs	Median	Range
Lung-ITV V_20 Gy _(%)	3.39	(0.5-32.3)
Chest wall V_30 Gy_ (cc)	6.3	(0-109.5)

Disease outcomes

**Figure 1:**
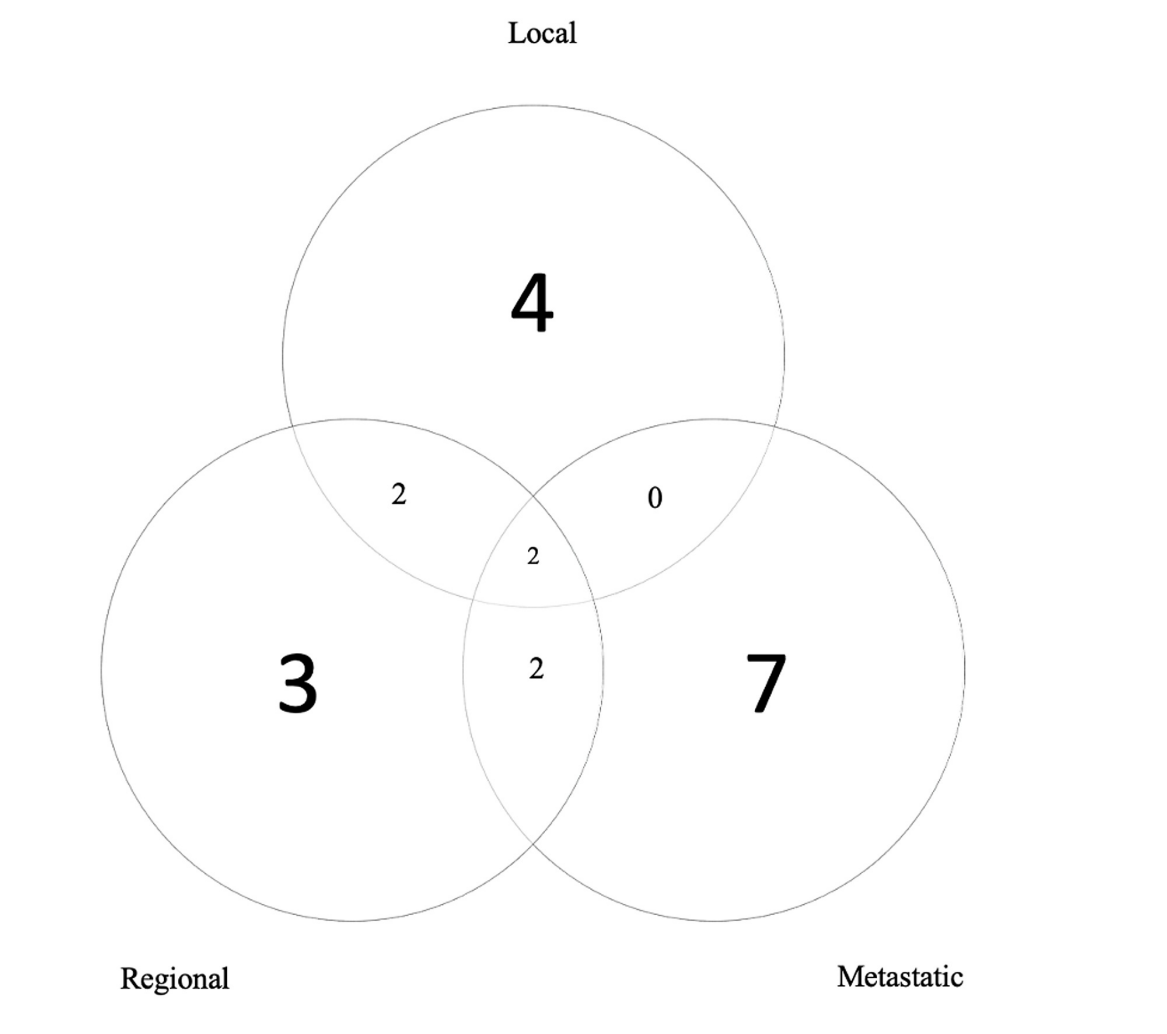
Patterns of failure.

**Figure 2:**
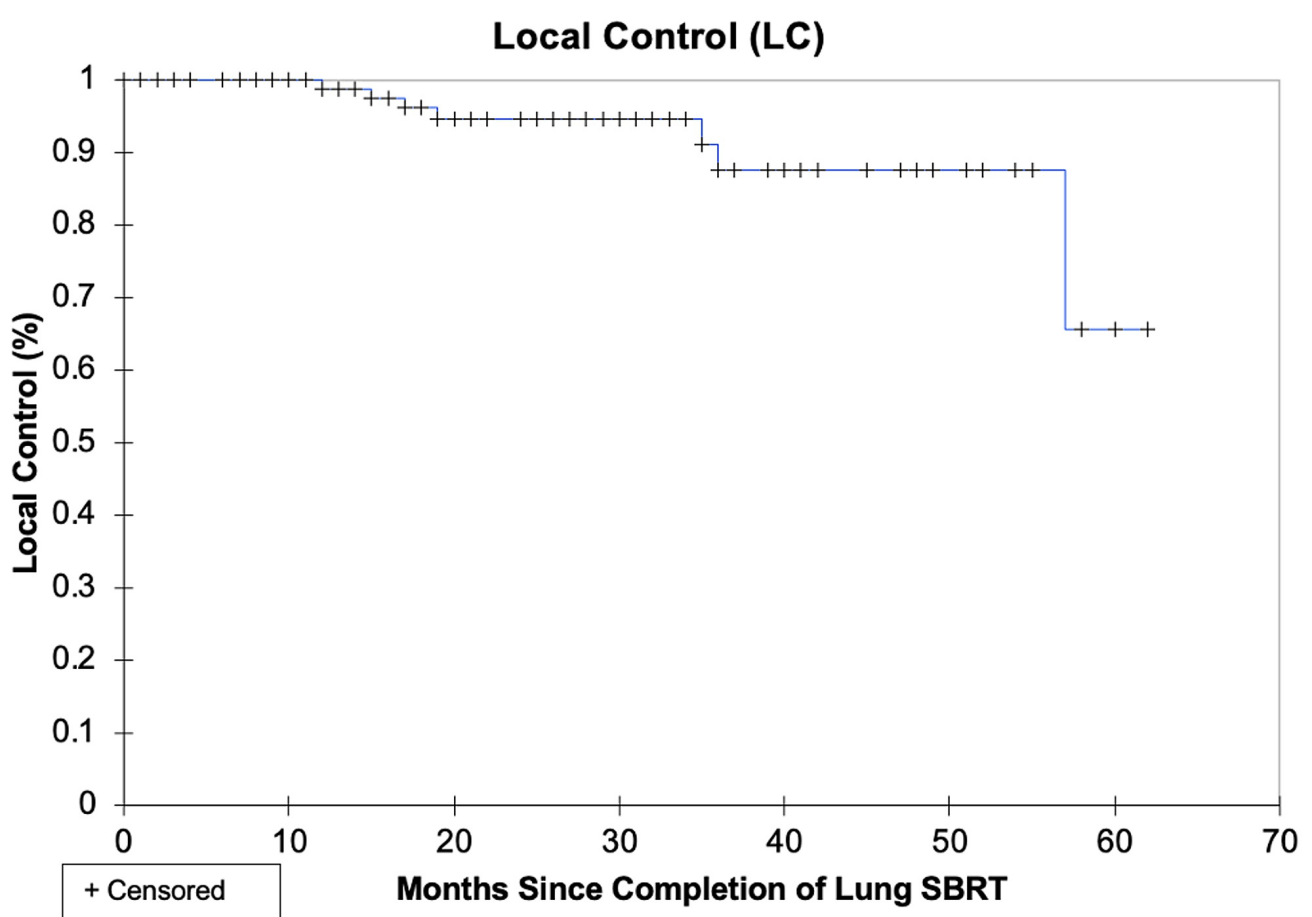
Kaplan-Meier local control (LC) curve. SBRT: stereotactic body radiation therapy.

**Figure 3:**
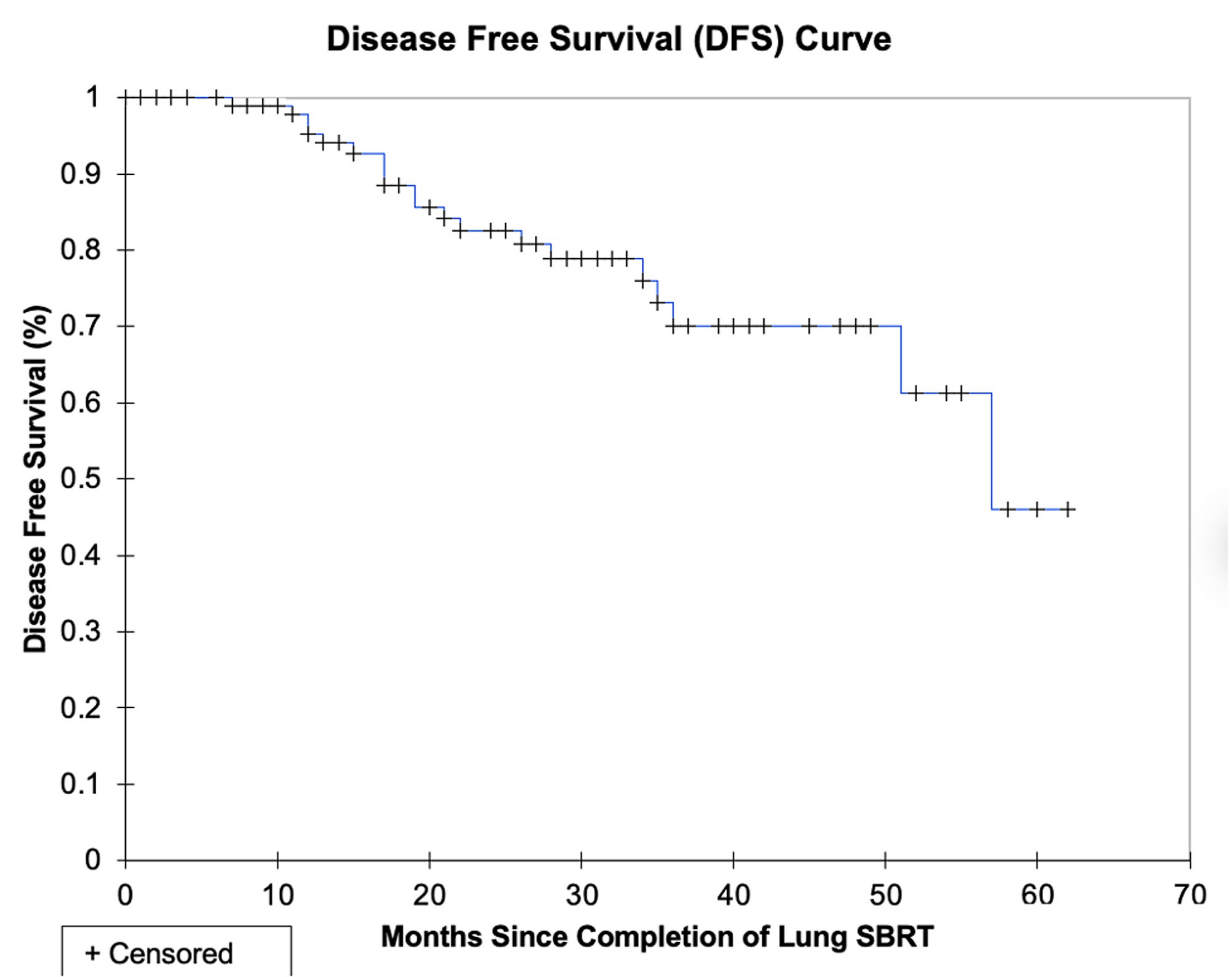
Kaplan-Meier disease-free survival (DFS) curve.

**Figure 4:**
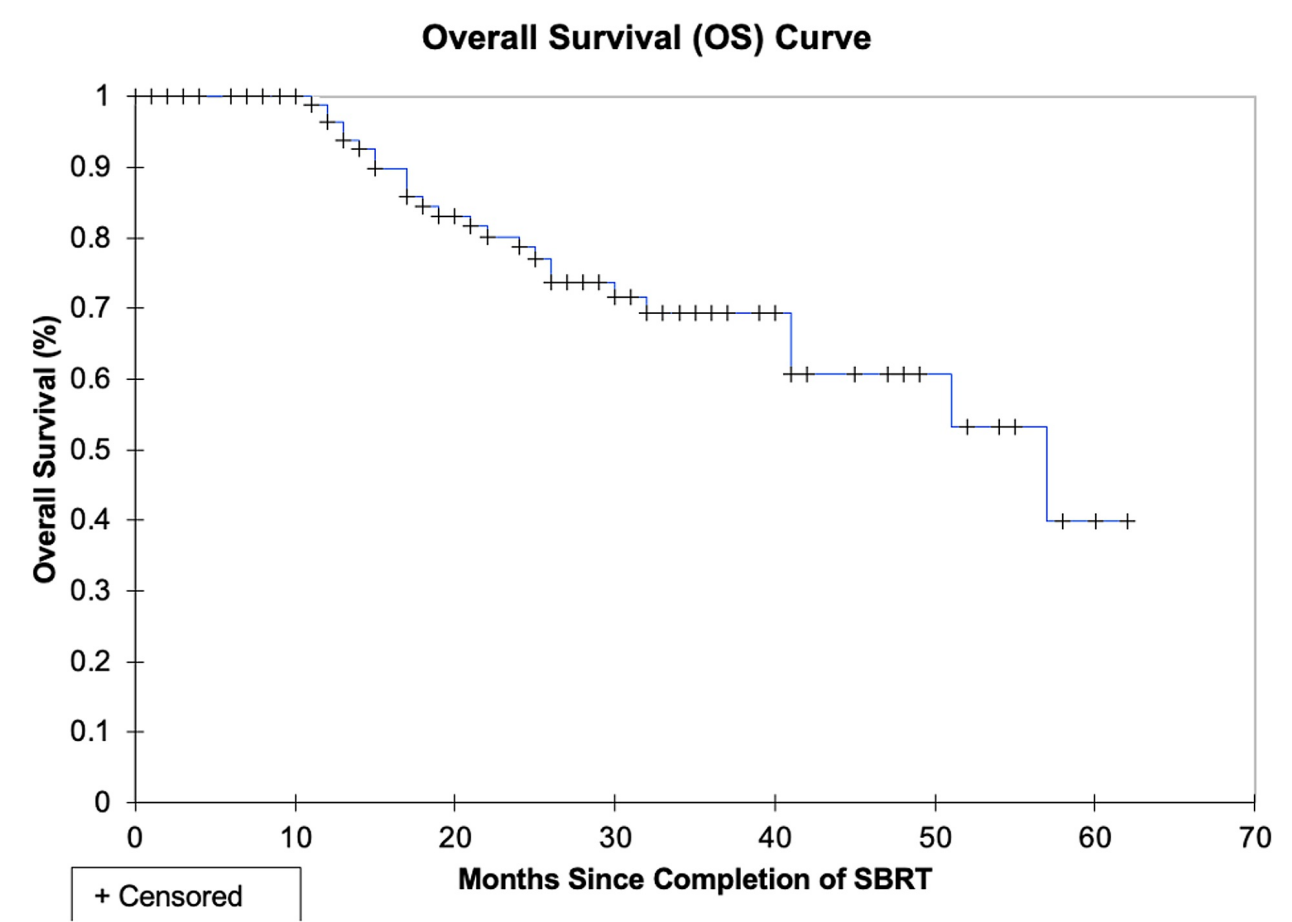
Kaplan-Meier overall survival (OS) curve. SBRT: stereotactic body radiation therapy.

The median survival was not reached and the two-year DFS and OS were 87.7% and 88.7%, respectively. The three-year DFS and OS were 81.0% and 80.0%, respectively. The local, lobar, regional, locoregional, and disseminated recurrence rates for individual lesions were 4.8%, 4.8%, 3.8%, 5.7%, and 10.5%, respectively. The rate of local control (defined as no intralobar failure) was 92.4% at three years. The pattern of relapse amongst all patients included 7 local failures, 12 regional failures, and 10 distant failures; these patterns of failure are shown pictorially in Figure 1. These data are summarized in Table 2. LC, DFS, and OS are represented in Kaplan-Meier curves in Figures 2, 3, 4, respectively.

**Table 2:** Disease outcomes, as compared to prospective NRG/RTOG trials. WRNMMC: Walter Reed National Military Medical Center; LC: local control; DFS: disease-free survival; OS: overall survival; RTOG: Radiation Therapy Oncology Group.

	WRNMMC	Grills, 2010	RTOG 0236	RTOG 0236	STARS/ROSEL	RTOG 0618
Patients (n)	105	55	55	55	31	26
Median follow-up length (months)	24	30	39	60	40	48
Recurrence						
Local	5 (4.8%)	2 (4%)	1 (2%)	4 (7%)	1 (3%)	1 (4%)
Lobar	5 (4.8%)		3 (5%)	9 (16%)		0
Regional	4 (3.8%)	2 (4%)	2 (4%)	7 (13%)	4 (13%)	3 (12%)
Locoregional	6 (5.7%)	5 (9%)	11%	38%		3 (12%)
Disseminated	12 (10.5%)	10 (18%)	11 (22%)	15 (31%)	1 (3%)	5 (19%)
LC	92.4 %	92%	90.6%	80.0%	96%	96%
DFS (%)	81.0 %	93%	48.30%	26%	86%	57%
OS (%)	80.0 %	72%	55.80%	40%	95%	56%

21.9% of our patients developed Grade 1 toxicity, largely chest wall pain, asymptomatic pneumonitis, and/or dyspnea. 6.7% of patients developed Grade 2 toxicity with a slight increase in dyspnea as well as a single mildly symptomatic rib fracture, not requiring intervention between non-opioid analgesics. No Grade 3 or higher was noted in our cohort. These data are summarized in Table 3. These are in accordance with CTCAE v5.0.

**Table 3:** Treatment-related toxicity, as per CTCAE v5.0. CTCAE: Common Terminology Criteria for Adverse Events.

	Grade 1	Grade 2	Grade 3-5	Total
Chest wall pain	9 (8.5%)	1 (0.9%)	0	10 (9.4%)
Pneumonitis	6 (5.7%)	1 (0.9%)	0	7 (6.6%)
Dyspnea	8 (7.5%)	4 (3.8%)	0	12 (11.3%)
Rib fracture	0	1 (0.9%)	0	1 (0.9%)

Biopsy versus non-biopsy

**Figure 5:**
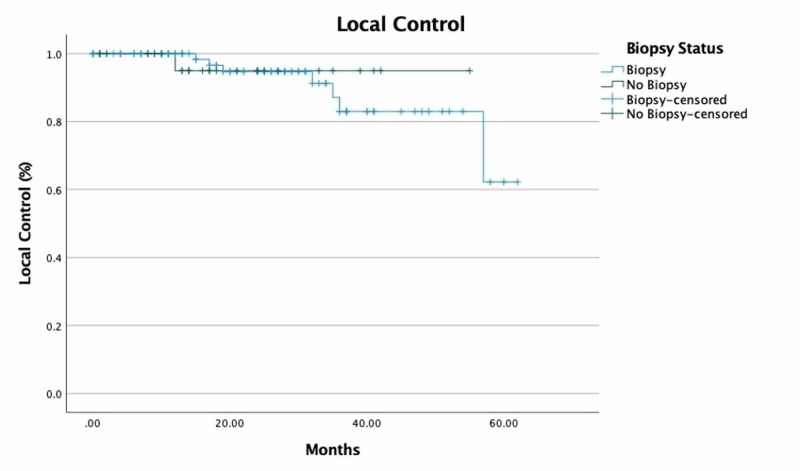
Local control, biopsy versus non-biopsy.

**Figure 6:**
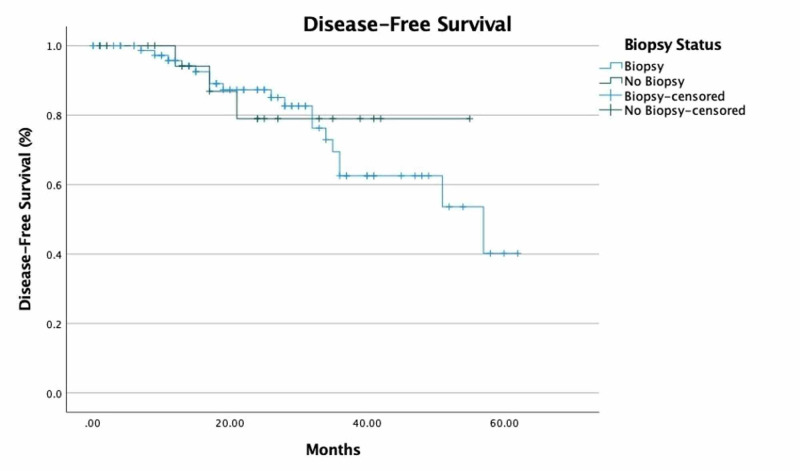
Disease-free survival, biopsy versus non-biopsy.

**Figure 7:**
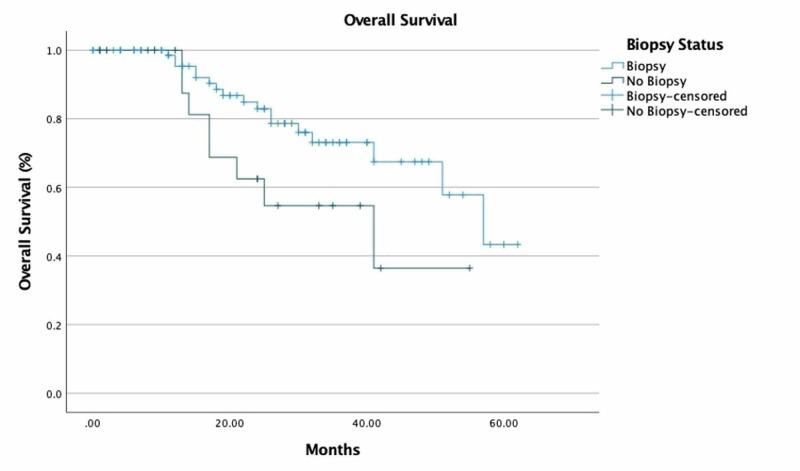
Overall survival, biopsy versus non-biopsy.

We performed a comparison of patients who had a biopsy vs. those who did not have a biopsy (or an inconclusive biopsy). There was no difference with respect to any demographic or treatment characteristic, including age, smoking history, or age (see Table 5 in Appendix). We also compared the rates of LC (p=0.75) and DFS (p=0.69) with no significant difference between both cohorts. In the comparison of overall survival, there was a trend (p=0.06) towards improved survival in the biopsy cohort. These data are depicted on Kaplan-Meier curves in the Appendix (Figures 5, 6, 7).

## Discussion

Our report is the first in the existing literature to compare lung SBRT for early-stage NSCLC in a non-academic setting to prospective trials conducted at large academic medical centers. It is also among the earliest and largest retrospective reports of lung SBRT disease outcomes and treatment toxicity in the non-academic setting with integrated and routine multidisciplinary management. It is also one of the first to report lung SBRT outcomes in an equal-access population with universal health care coverage [11].

The treatment of lung cancer in the non-academic setting may present numerous challenges to providing equitable disease control outcomes. There may exist many challenges to screening that include access to care, awareness of screening options, and implicit bias due to stigmatization of smoking that has been reported in patient experiences [12]. Additionally, there has been reported increased difficulty of shared decision making due to these challenges [12]. Lung cancer patients with decreased access to primary care have been reported to have inferior survival [8]. Practicing in the community may also pose unique challenges such as geographical distance to care and sociodemographic divides that have also been associated with inferior survival in lung cancer patients [13,14]. While practicing in the non-academic setting, our institution has taken advantage of its universal access to care and implemented multiple measures to ensure that such community barriers may be overcome. These include comprehensive primary care, a robust lung cancer screening program, routine follow-up, and fully integrated multidisciplinary lung cancer treatment. These unique aspects of patient care at WRNMMC may be applicable in other community settings so that lung SBRT outcomes comparable to prospective trials at large academic medical centers may be achieved.

In order to better understand the basis of comparison, it is necessary to compare our outcomes to the various prospective trials (summarized in Table 2). Grills et al. reported on the William Beaumont single-institution experience in a prospective, non-randomized analysis comparing patients who received lung SBRT versus surgical resection. There were 58 patients in the radiation cohort treated between 2003 and 2009, with a median follow-up of 30 months. Patients received either 48 Gy in 4 fractions or 60 Gy in 5 fractions [15]. Local, regional, and locoregional recurrence were 4%, 4%, and 9% [15], respectively, and were similar to our findings. The rate of disseminated failure was 18% [15], slightly higher than our rate of 10.5%. Rates of LC were very similar at 92%, the overall range of DFS and OS of 93% and 72% [15], respectively, are similar to our findings of 81% and 80%, respectively. This was one of the earliest prospective reports of lung SBRT and has largely similar disease control findings to our cohort.

STARS-ROSEL was a pooled analysis of two independent but small randomized studies comparing surgery with lung SBRT, both of which closed early due to slow accrual [16]. The primary endpoint was OS, and 31 patients were randomized to the SBRT arms between 2008 and 2013 with a median follow-up length of 40.2 months [16]. Patients received either 54 Gy in 3 fractions or 50 Gy in 4 fractions, based on peripheral or central location, respectively [16]. The reported rates of local, regional, and disseminated recurrence were 3%, 13%, and 3% [16], respectively, which are similar to our data with respect to locoregional control but lower in the rate of disseminated recurrence. They also reported excellent disease outcomes of LC, DFS, and OS rates of 96%, 86%, and 95% [16], respectively. It is unclear if these rates appear more favorable than our patient cohort due to either patient selection (both studies closed due to slow accrual) or some other unseen factor. Overall, Grills et al. and STARS-ROSEL had similar rates of disease control and survival outcomes, likely due to a similar length of follow-up. These comparisons indicate that we were able to experience similar disease outcomes in our military non-academic setting to these prospective trials conducted at large academic centers with similar durations of follow-up.

RTOG 0236 and 0618 were both multicenter, single-arm, phase 2 trials using SBRT for early-stage, medically inoperable NSCLC. RTOG 0236 reported initial results in 2010 [17] and subsequently updated in 2018 [4]. Fifty-five patients were followed for a median time of 48 months, and all were treated with 60 Gy in 3 fractions (later amended with heterogeneity corrections to 54 Gy in 3 fractions) between 2004 and 2006 [4]. In their first report at a median follow-up of 39 months, rates of disease recurrence for local, lobar, regional, locoregional, and disseminated were 2%, 5%, 4%, 11%, and 22% [17]; these are similar to our rates of recurrence. However, while their LC was comparable at 90.6% in their initial report, the rates of DFS and OS were 48.3% and 55.8% [17] respectively, were notably lower than in our cohort. This is likely due to the fact that they had a longer duration of follow-up and these patients were treated nearly 10-15 years prior to our cohort, as lung cancer survival has improved in that timeframe [18,19]. This is further confirmed by their updated report on their data at a median follow-up of 60 months, with increased rates of disease recurrence and inferior survival outcomes [4].

RTOG 0618 was a very similar trial that enrolled a total of 26 patients between 2007 and 2010 [3]. Patients received 54 Gy in 3 fractions [3]. At a median follow-up duration of 48 months, their rates of disease recurrence for local, lobar, regional, locoregional, and disseminated were 4%, 0%, 12%, 12%, and 19% [3]. While these rates are higher than in our cohort, they had a significantly longer follow-up period. This is also reflected in the rates of DFS and OS of 57% and 56% respectively, despite excellent LC at 96% [3]. These findings are similar with RTOG 0236, where the rates of recurrence and survival appear inferior to our cohort, but this is likely due to an earlier treatment time period and a longer period of follow-up.

With respect to treatment-related toxicity, we report an excellent toxicity profile with reasonable Grade 1 (21.9%) and 2 (6.7%) toxicity, and no Grade 3 or higher toxicity. Grills et al. were the only out of these prospective trials to report Grade 1 and 2 toxicity, with higher Grade 1 toxicity (38%) but similar Grade 2 toxicity (9%), when compared to our cohort [15]. Grade 1 or 2 toxicity was not reported in the other prospective studies [3,4,16]. Grade 3 toxicity was highly variable in reported rates in these trials even across varying lengths of follow-up, ranging from 2% in Grills et al. [15] to 32.3% in STARS/ROSEL [16]. Out of all the prospective trials, only RTOG 0236 reported any Grade 4 toxicity (3.6%) [4]. These data are summarized in Table 4. On the whole, the favorable toxicity profile of lung SBRT overall appears consistent between our studies and other prospective trials. Our toxicity analysis for low-grade treatment toxicities is better reported than most of these prospective series. However, an in-depth analysis of our treatment toxicity and its full clinical impact may be limited by the shorter length of follow-up in our cohort.

**Table 4:** Treatment-related toxicity, as compared to recent prospective series per CTCAE v5.0. CTCAE: Common Terminology Criteria for Adverse Events; WRNMMC: Walter Reed National Military Medical Center; RTOG: Radiation Therapy Oncology Group.

	Grade 1	Grade 2	Grade 3	Grade 4	Grade 5
WRNMMC	23 (21.9%)	7 (6.7%)	0	0	0
Grills et al, 2010 [15]	21 (38%)	5 (9%)	1 (2%)	0	0
RTOG 0236 [17]	N/A	N/A	4 (7.3%)	0	0
RTOG 0236 [4]	N/A	N/A	15 (27.3%)	2 (3.6%)	0
STARS/ROSEL [16]	N/A	N/A	10 (32.3%)	0	0
RTOG 0618 [3]	N/A	N/A	2 (7.7%)	0	0

There exist two prior large retrospective series to report on lung SBRT outcomes in the non-academic setting, without equal access to care and routine integration of multidisciplinary care. Davis et al. reported on a multi-institutional patient registry on 723 patients with a median follow-up of 12 months with long-term LC, DFS, and OS of 87%, 81.6%, and 60%, respectively; they did not report on treatment-related toxicity [20]. Due to the many institutions included, there was large variability in the dose/fractionation of SBRT, and so BED was used to compare various disease outcomes. Heal et al. reported on a single-institution experience of 100 patients with a median follow-up of 27.5 months, with long-term LC and OS were 84.3% and 37.2% respectively; DFS was not reported [21]. They reported 1 (1%) Grade 3 toxicity, with no grade 4 or higher toxicity seen [21]. Our data showed similar results in terms of these disease outcomes and treatment toxicity, with a similar length of follow-up. While our data came from a single institution, the standardization of dose/fractionation and practice patterns allows for more generalizability of our findings within a community setting. On the whole, we also reported more disease outcome-related endpoints as well as more comprehensive treatment toxicity reporting than these two retrospective studies. Additionally, our report is the first to highlight these data in the non-academic setting in a patient population with decreased barriers to care, with universal coverage and routine multidisciplinary management.

Prior studies have established lung cancer patients in an equal access military healthcare system (MHS) are able to overcome large disparities in receipt of treatment and treatment outcomes, such as race [22] and preexisting mental disorders [23]. The MHS is also unique as the scope of practice is within the non-academic setting, however, all patients at WRNMMC undergo comprehensive multidisciplinary evaluation by largely fellowship-trained subspecialists. We have incorporated key components of setting up a multidisciplinary lung cancer effort in a community setting that have previously been identified including: weekly tumor board, thoracic surgeons with minimally-invasive surgery experience, utilization of treatment guidelines, formal continuing medical education, emphasis on lung cancer screening protocols, and a single location of care [24,25]. Previous reports highlight the benefits derived from establishing such a program: significant improvements in the quality of care (including decreased time from diagnosis to treatment initiation), patient satisfaction and retention of patients [26]. Our favorable reported disease outcomes and toxicity show that in a non-academic setting, factors such as equal access to care and routine multidisciplinary evaluation can help ensure excellent lung cancer outcomes.

Our report is the first non-academic retrospective series to include a large proportion of patients (36.8%) who did not have biopsy-proven confirmation of diagnosis; it should be noted that a third (13/39) of this subset of patients did have at least an attempt at biopsy with inconclusive findings. All the prospective series and several recent large community-based retrospective series discussed above included only patients with the biopsy-proven disease. In order to eliminate confounding factors in our comparisons, we compared treatment characteristics, demographics, as well as disease outcomes in biopsy vs. non-biopsy cohorts, with no significant differences between cohorts (see Appendix). Of note, there was a trend towards improved overall survival in the biopsy-cohort (p=0.06), which is likely reflective of this cohort's superior baseline performance status and expected life expectancy.

It must be noted that invasive lung diagnostic procedures are associated with complication rates in 22%-24% of cases [27], and this rate may be even higher in the lung SBRT patient population who often have multiple comorbidities. National-level trends suggest empiric treatment with lung SBRT without a biopsy has increased over time and will likely continue to increase [28]. Accordingly, there is also a growing body of guidelines that support clinical decision-making of treatment of suspicious lesions empirically without a biopsy [29]. Additionally, a recent retrospective case-matched cohort analyses for 701 patients (1/3 not biopsied) were compared in biopsy versus no-biopsy cohorts without any significant differences in disease outcomes, further corroborating that empiric treatment is routine and safe [30]. In our non-academic cancer center, all patients must have full multidisciplinary tumor board buy-in prior to treatment without biopsy confirmation. While this could lead to higher reported rates of disease control, there is a vast body of literature that supports this contemporary norm in the treatment of lung SBRT.

This observational study is limited by its single-institution retrospective design and is prone to selection biases with regard to patient treatment, patient fitness, and loss of patient follow-up (due to death and/or comorbid illnesses). Moreover, limitations exist regarding tracking toxicity events by review of the electronic medical record, and there is a small risk of underestimation of these rates of toxicity. Despite these limitations, we believe these results are a valuable addition to the literature to highlight early-stage NSCLC outcomes after SBRT in a military setting.

Due to the lack of public availability of the data in the prospective trials, our data was only empirically and qualitatively compared to the prospective data rather than in a statistically definitive manner. Furthermore, the heterogeneity of these prospective trials with varying endpoints may limit our ability to compare our data with these trials. It should also be noted that nearly all the prospective comparison cohorts were treated nearly 5-10 years before the time period of when our patients were treated, and there may be advents in radiation technology that could translate into varied outcomes and toxicity profiles of lung SBRT. Additionally, many of the earlier prospective trials were conducted using different LINAC platforms (e.g., CyberKnife) with different levels of image guidance than in our retrospective cohort treated on a LINAC-based system. However, it should be noted that the widespread availability to convert conventional LINACs to be able to perform volume modulated arc therapy (VMAT)-based SBRT is part of its improved accessibility in the community setting.

Nearly 40% of our patients lacked definitive biopsy-confirmed disease, with potential uncertainty regarding a large proportion of treated lesions. However, patients were only treated after multidisciplinary tumor board evaluation and consensus that the lesion had a significantly high likelihood of representing malignancy, based on serial imaging and PET avidity. We also compared biopsy vs non-biopsy cohorts without any significant differences in any potential confounding factors related to treatment characteristics of disease outcomes.

Further investigation into prospective trials targeted in the non-academic setting is warranted. With no shortage of data from high-volume academic centers, prospective data from this setting will help to better understand the ideal implementation of lung SBRT for patients who may face greater barriers to care. Furthermore, comparing early-stage lung cancer outcomes after SBRT in an equal access setting warrants prospective investigation to see how barriers to care in the community setting can be overcome, so they may be widely applicable.

## Conclusions

Our report is the first in the existing literature to compare lung SBRT for early-stage NSCLC in the non-academic setting with prospective trials conducted at large academic centers. We observed excellent disease outcomes and treatment toxicity outcomes consistent with recently published prospective data. Given previous reports showing disparities in cancer care and outcomes in the community setting for lung cancer, these findings suggest an equal access system can provide high-quality multidisciplinary care to help overcome these disparities.

## Appendices

**Table 5:** Demographics and patient characteristics, biopsy versus non-biopsy. pk/yr: pack-years; PFTs: pulmonary function tests; FEV1: forced expiratory volume in one second; DLCO: diffusing capacity for carbon monoxide; SCC: squamous cell carcinoma; PTV: planning target volume; fx: fractions; OAR: organ-at-risk; ITV: internal target volume.

	Biopsy	No biopsy	p-Value
Sex	n	n	
Male	48	14	0.950
Female	40	12	
Age	Median	Median	
(years)	77	77	0.342
Smoking	n	n	
<10 pk/yr	16	7	0.133
10-39 pk/yr	21	9	
40 or more pk/yr	51	10	
PFTs (%)	Median	Median	
FEV 1	79	80	0.079
DLCO	57	60	0.462
Stage	n	n	
T1	63	20	0.334
T2	20	3	
T3	3	1	
Size	Median	Median	
(cm)	1.85	1.6	0.055
PTV	Median	Median	
(cc)	25.91	15.55	0.083
Dose/# Fractions	n	n	
50 Gy/ 5 fx	75	23	0.438
54 Gy/ 3 fx	4	0	
60 Gy/ 8 fx	9	3	
Dmax	Median	Median	
(%)	123.9	123.5	0.886
Organs at Risk	Median	Median	
Lung-ITV V_20 Gy _(%)	3.58	2.53	0.401
Chest wall V_30 Gy_ (cc)	8.95	4	0.169

## References

[1] Ball D, Mai GT, Vinod S (2019). Stereotactic ablative radiotherapy versus standard radiotherapy in stage 1 non-small-cell lung cancer (TROG 09.02 CHISEL): a phase 3, open-label, randomised controlled trial. Lancet Oncol.

[2] Nyman J, Hallqvist A, Lund JÅ (2016). SPACE - A randomized study of SBRT vs conventional fractionated radiotherapy in medically inoperable stage I NSCLC. Radiother Oncol.

[3] Timmerman RD, Paulus R, Pass HI (2018). Stereotactic body radiation therapy for operable early-stage lung cancer: findings from the NRG Oncology RTOG 0618 Trial. JAMA Oncol.

[4] Timmerman RD, Hu C, Michalski JM (2018). Long-term results of stereotactic body radiation therapy in medically Iinoperable stage I non-small cell lung cancer. JAMA Oncol.

[5] Patel AP, Crabtree TD, Bell JM (2014). National patterns of care and outcomes after combined modality therapy for stage IIIA non-small-cell lung cancer. J Thorac Oncol.

[6] Ramalingam S, Dinan MA, Crawford J (2018). Survival comparison in patients with stage IV lung cancer in academic versus community centers in the United States. J Thorac Oncol.

[7] Samson P, Patel A, Crabtree TD (2015). Multidisciplinary Treatment for Stage IIIA Non-Small Cell Lung Cancer: Does Institution Type Matter. Ann Thorac Surg.

[8] Su CT, Chau V, Halmos B (2019). Impact of primary care access on mortality of lung cancer patients in an underserved community. Am J Clin Oncol.

[9] Koshy M, Malik R, Spiotto M, Mahmood U, Weichselbaum R, Sher D (2015). Disparities in treatment of patients with inoperable stage I non-small cell lung cancer: a population-based analysis. J Thorac Oncol.

[10] Lin J, Kamamia C, Brown D (2018). Survival among lung cancer patients in the U.S. Military Health System: a comparison with the SEER population. Cancer Epidemiol Biomarkers Prev.

[11] Bryant AK, Mundt RC, Sandhu AP (2018). Stereotactic body radiation therapy versus surgery for early lung cancer among US veterans. Ann Thorac Surg.

[12] Borondy Kitts AK (2019). The patient perspective on lung cancer screening and health disparities. J Am Coll Radiol.

[13] Osuoha CA, Callahan KE, Ponce CP, Pinheiro PS (2018). Disparities in lung cancer survival and receipt of surgical treatment. Lung Cancer.

[14] Ellis L, Canchola AJ, Spiegel D, Ladabaum U, Haile R, Gomez SL (2018). Racial and ethnic disparities in cancer survival: the contribution of tumor, sociodemographic, institutional, and neighborhood characteristics. J Clin Oncol.

[15] Grills IS, Mangona VS, Welsh R (2010). Outcomes after stereotactic lung radiotherapy or wedge resection for stage I non-small-cell lung cancer. J Clin Oncol.

[16] Chang JY, Senan S, Paul MA (2015). Stereotactic ablative radiotherapy versus lobectomy for operable stage I non-small-cell lung cancer: a pooled analysis of two randomised trials. Lancet Oncol.

[17] Timmerman R, Paulus R, Galvin J (2010). Stereotactic body radiation therapy for inoperable early stage lung cancer. JAMA.

[18] Xia W, Yu X, Mao Q (2017). Improvement of survival for non-small cell lung cancer over time. Onco Targets Ther.

[19] Dillman RO, McClure SE (2014). Steadily improving survival in lung cancer. Clin Lung Cancer.

[20] Davis JN, Medbery C, Sharma S (2015). Stereotactic body radiotherapy for early-stage non-small cell lung cancer: clinical outcomes from a National Patient Registry. J Radiat Oncol.

[21] Heal C, Ding W, Lamond J (2015). Definitive treatment of early-stage non-small cell lung cancer with stereotactic ablative body radiotherapy in a community cancer center setting. Front Oncol.

[22] Williams CD, Salama JK, Moghanaki D, Karas TZ, Kelley MJ (2016). Impact of race on treatment and survival among U.S. veterans with early-stage lung cancer. J Thorac Oncol.

[23] Lin J, McGlynn KA, Carter CA (2016). The impact of preexisting mental health disorders on the diagnosis, treatment, and survival among lung cancer patients in the U.S. military health system. Cancer Epidemiol Biomarkers Prev.

[24] Folman RS (1990). Multidisciplinary management of non-small cell lung cancer in the community. Semin Oncol.

[25] Fischel RJ, Dillman RO (2009). Developing an effective lung cancer program in a community hospital setting. Clin Lung Cancer.

[26] Bjegovich-Weidman M, Haid M, Kumar S, Huibregtse C, McDonald J, Krishnan S (2010). Establishing a community-based lung cancer multidisciplinary clinic as part of a large integrated health care system: aurora health care. J Oncol Pract.

[27] Huo J, Xu Y, Sheu T, Volk RJ, Shih YT (2019). Complication rates and downstream medical costs associated with invasive diagnostic procedures for lung abnormalities in the community setting. JAMA Intern Med.

[28] Rutter CE, Corso CD, Park HS (2014). Increase in the use of lung stereotactic body radiotherapy without a preceding biopsy in the United States. Lung Cancer.

[29] Videtic GMM, Donington J, Giuliani M (2017). Stereotactic body radiation therapy for early-stage non-small cell lung cancer: Executive Summary of an ASTRO Evidence-Based Guideline. Pract Radiat Oncol.

[30] Fernandez C, Grills IS, Ye H (2020). Stereotactic image guided lung radiation therapy for clinical early stage non-small cell lung cancer: a long-term report from a multi-institutional database of patients treated with or without a pathologic diagnosis. Pract Radiat Oncol.

